# A randomized Phase 2 trial of telavancin versus standard therapy in patients with uncomplicated *Staphylococcus aureus* bacteremia: the ASSURE study

**DOI:** 10.1186/1471-2334-14-289

**Published:** 2014-05-23

**Authors:** Martin E Stryjewski, Arnold Lentnek, William O’Riordan, John Pullman, Paul Anantharajah Tambyah, Jose M Miró, Vance G Fowler Jr, Steven L Barriere, Michael M Kitt, G Ralph Corey

**Affiliations:** 1 Department of Medicine, Division of Infectious Diseases, Centro de Educación Médica e Investigaciones Clinicas (CEMIC) “Norberto Quirno”, Av. Las Heras 2981, Office #3, Capital Federal (C1425ASG), Buenos Aires, Argentina; 2 Wellstar Infectious Disease, Marietta GA, USA; 3 eStudy Site, San Diego CA, USA; 4 Mercury Street Medical Group, Butte MT, USA; 5 Department of Medicine, National University Health System, Singapore, Singapore; 6 Infectious Diseases Service, Hospital Clinic–Institut d’investigacions Biomèdiques August Pi i Sunyer, University of Barcelona, Barcelona, Spain; 7 Duke Clinical Research Institute, Durham NC, USA; 8 Department of Medicine, Division of Infectious Diseases, Duke University Medical Center, Durham NC, USA; 9 Theravance, Inc, South San Francisco CA, USA

**Keywords:** Bacteremia, *Staphylococcus aureus*, Telavancin, Vancomycin

## Abstract

**Background:**

*Staphylococcus aureus* bacteremia is a common infection associated with significant morbidity and mortality. Telavancin is a bactericidal lipoglycopeptide active against Gram-positive pathogens, including methicillin-resistant *S. aureus* (MRSA). We conducted a randomized, double-blind, Phase 2 trial in patients with uncomplicated *S. aureus* bacteremia.

**Methods:**

Patients were randomized to either telavancin or standard therapy (vancomycin or anti-staphylococcal penicillin) for 14 days. Continuation criteria were set to avoid complicated *S. aureus* bacteremia. The primary end point was clinical cure at 84 days.

**Results:**

In total, 60 patients were randomized and 58 received ≥1 study medication dose (all-treated), 31 patients fulfilled inclusion/exclusion and continuation criteria (all-treated target [ATT]) (telavancin 15, standard therapy 16), and 17 patients were clinically evaluable (CE) (telavancin 8, standard therapy 9). Mean age (ATT) was 60 years. Intravenous catheters were the most common source of *S. aureus* bacteremia and ~50% of patients had MRSA. A similar proportion of CE patients were cured in the telavancin (88%) and standard therapy (89%) groups. All patients with MRSA bacteremia were cured and one patient with MSSA bacteremia failed study treatment in each group. Although adverse events (AEs) were more common in the telavancin ATT group (90% vs. 72%), AEs leading to drug discontinuation were similar (7%) in both treatment arms. Potentially clinically significant increases in serum creatinine (≥1.5 mg/dl and at least 50% greater than baseline) were more common in the telavancin group (20% vs. 7%).

**Conclusions:**

This study suggests that telavancin may have utility for treatment of uncomplicated *S. aureus* bacteremia; additional studies are warranted. (Telavancin for Treatment of Uncomplicated *Staphylococcus Aureus* Bacteremia (ASSURE); NCT00062647).

## Background

*Staphylococcus aureus* bacteremia (SAB) is a devastating infection associated with high mortality, morbidity, and medical costs [1-3]. Approximately 40% of patients with SAB will have a complicated infection and more than 25% of these patients will die within the 12 weeks following their initial positive blood culture [2]. Bacteremia is also a common form of invasive infection due to methicillin-resistant *S. aureus* (MRSA) [4].

Vancomycin, the most commonly used antibiotic in hospitalized patients with MRSA bacteremia, has several limitations [5], including the rise of strains with decreased susceptibility [6,7] or resistance [8,9] and suboptimal results for the treatment of patients with bacteremia due to methicillin-susceptible *S. aureus* (MSSA) [10,11]. Despite the dramatic consequences of SAB and the limitations of vancomycin, only one registrational, open-label, randomized clinical trial in patients with SAB has been conducted [12], and vancomycin remains first-line therapy for MRSA bacteremia in most settings [13].

Telavancin is a lipoglycopeptide antibacterial agent exhibiting concentration-dependent bactericidal effects via a dual mechanism of action that combines inhibition of cell wall synthesis and disruption of membrane barrier function [14-16]. Telavancin has been approved in the U.S. and Canada for the treatment of patients with complicated skin and skin structure infections due to Gram-positive pathogens, and in Europe for the treatment of hospital-acquired pneumonia (including ventilator-associated pneumonia) due to MRSA, when alternative medicines are unsuitable^a^. Most recently (June 2013), telavancin was approved in the U.S. for hospital-acquired and ventilator-associated bacterial pneumonia caused by susceptible isolates of *S. aureus* when alternative treatments are not suitable. *In vitro*, telavancin is bactericidal against clinically important Gram-positive bacteria, including MSSA, MRSA, vancomycin intermediate susceptible (VISA) [17], and hetero-intermediate strains (hVISA) [18]. Animal models of infection suggested that telavancin may be an effective treatment for SAB and endocarditis [17,19,20]. However, clinical experience in patients with SAB or endocarditis due to *S. aureus* treated with telavancin is limited [21,22].

The ASSURE study (Telavancin for Treatment of Uncomplicated *S. aureus* Bacteremia) was conducted as a proof-of-concept study for telavancin compared with standard therapy for the treatment of uncomplicated SAB.

## Methods

This proof-of-concept study was a Phase 2, randomized, double-blind, active-controlled, parallel group, multinational trial (NCT00062647), conducted from August 2003 through August 2006. Studies were approved by each institutional review board or ethics committee (see Additional file 1), and written informed consent was obtained from all patients or their legal representatives. Patients were randomized through an interactive voice response system in a 1:1 ratio, using a permuted block algorithm. The randomization was stratified by geographic region (within or outside the U.S.).

### Study population and continuation criteria

Patients were considered for the study if they were ≥18 years of age and had uncomplicated *S. aureus* bacteremia (with a qualifying blood culture [QBC]). Patients were excluded if they had any of the following: non-removable hardware (e.g., vascular stents or grafts placed within the last 6 weeks, joint prosthesis, or cardiovascular devices), removable source of infection (e.g., central catheter) that was not planned to be removed within 24 h from the QBC, significant cardiac valvular disease, any intra-cardiac mass or abscess defined by transthoracic or transesophageal echocardiography, prior history of endocarditis or osteomyelitis, recent infection with *S. aureus* requiring systemic antibacterial therapy within the last 30 days, evidence of metastatic complication (e.g., deep abscesses, endocarditis, osteomyelitis), or signs of vascular phenomena indicating potential arterial embolism (e.g., brain hemorrhage, pulmonary infarcts, Janeway lesions). Patients were also excluded if they had QTc (Fredericia’s corrected) >470 msec, uncompensated heart failure or unstable angina within the last 30 days, abnormal serum levels of potassium or magnesium that could not be corrected, hypotension or oliguria unresponsive to resuscitation with fluid or vasopressors, recent systemic antimicrobial therapy potentially effective against *S. aureus* (>72 h within the last 7 days), neutrophil count <500 cells/mm^3^, HIV infection with CD4 count <100 cells/mm^3^ during the last 6 months, alanine aminotransferase or aspartate aminotransferase >5-fold the upper limit of normal or with Child-Pugh class B or C hepatic disease, immunosuppressant therapy, or concomitant use of agents containing cyclodextrin.

After initiation of study drug, patients were continued in the study only if they met all the following criteria: a) patients with MRSA bacteremia had been receiving vancomycin as initial therapy, b) removal of all removable foci within 24 h after the report of the QBC, c) negative follow-up blood cultures (FUBC) drawn 24 to 48 h after the report of the QBC, d) resolution of fever (≤38.0°C) within 72 h of the initiation of antistaphylococcal therapy, e) pre-treatment urine culture negative for *S. aureus*, f) transthoracic or transesophageal echocardiography, following QBC, showing no significant valvular disease, and g) no evidence of a metastatic complication on or before Day 5.

### Antimicrobial therapy

Patients were randomized to either telavancin 10 mg/kg intravenous (IV) q 24 h or standard therapy (vancomycin 1 g IV q 12 h, or nafcillin 2 g IV q 6 h, oxacillin 2 g IV q 6 h, or cloxacillin 2 g IV q 6 h). An anti-staphylococcal penicillin (ASP) could be selected if the baseline pathogen was known or highly suspected to be MSSA. The total duration of treatment with study medications was 14 days. Dummy infusions were used to maintain the study blinding and blinding of study medication was performed in the pharmacy.

Vancomycin was dosed per the U.S. Food and Drug Administration (FDA)-approved label. The dose of vancomycin could be adjusted by local institutionally accepted policies based on weight, serum levels, and/or renal function. Any dosage adjustments or obtention of serum levels of vancomycin were performed in a manner that maintained the study blind, usually by a designated pharmacist. Each site was to have submitted a blinding plan that was approved by the study sponsor.

The dose of telavancin was adjusted in patients with renal impairment: 7.5 mg/kg q 24 h and 10 mg/kg q 48 h for patients with creatinine clearance between 30 to 50 ml/min and <30 ml/min, respectively.

Vancomycin was continued in patients who were receiving vancomycin prior to randomization and were randomized to standard therapy. If the organism subsequently proved to be MSSA, vancomycin could have been changed to an ASP (nafcillin, oxacillin, or cloxacillin) at the investigator’s discretion.

### Assessments

Clinical assessments were conducted at baseline and daily through to the end of therapy (EOT). EOT evaluation was performed within 72 h after administration of the last dose of study medication. A follow-up (FU) visit was scheduled 7 to14 days after EOT, and a test-of-cure (TOC) visit was scheduled 84 days after the start of study medication. At each evaluation, investigators assessed the signs, symptoms, and extent of the infection, surgical procedures, adverse events (AEs), and concomitant medications.

Two independent blood culture (BC) specimens were obtained within 24 to 48 h after the report of the QBC, EOT, and at the FU visit. Blood cultures were not obtained at TOC or at other time points, unless clinically indicated. All pathogens isolated were sent to a central microbiology laboratory for identification of genus and species and MIC testing.

Electrocardiograms were obtained in triplicate at baseline, every third day, and at EOT evaluation. Laboratory tests were performed at baseline, every 3 days during treatment, and at EOT and FU visits.

### Study outcomes

Clinical response assessed by the investigators at TOC was the primary end point of the study. Cure was defined by all the following criteria: resolution of clinical symptoms/signs associated with the bacteremia, no evidence of metastatic complications, all cultures negative for *S. aureus* after QBC cultures, and no non-study systemic anti-staphylococcal medication to which the baseline pathogen was susceptible. Failure was defined by any of the following: presence of symptoms/signs associated with the bacteremia, evidence of metastatic complications, positive blood culture for *S. aureus*, or death related to initial study infection after study Day 3. Indeterminate was defined by the inability to determine the outcomes mentioned above.

### Analysis groups

The following groups were defined for the analysis: a) all-treated (AT), patients who received ≥1 dose of study medication, b) all-treated target (ATT) patients who received study medication and fulfilled all inclusion/exclusion and continuation criteria (or who were approved for inclusion after careful review by the medical monitor to determine that the patient could be appropriately assessed), and c) clinically evaluable (CE), patients in the ATT population who received 12 to 16 days of study medication and whose study participation did not deviate from the protocol by more than pre-specified limits.

The objective of this study was to assess safety, tolerability, and explore efficacy (proof-of-concept). Sample size (60 patients randomized) was selected on the basis of clinical judgment in order to provide informative results consistent with the study objective. This study was not designed or powered to produce statistically significant results. The difference between rates of response within each treatment group was estimated with 95% confidence interval (CI), calculated using the method of Agresti and Caffo [23].

The protocol was amended to increase the daily dose of telavancin to 12.5 mg/kg and to include patients with complicated bacteremia, allowing 28 to 42 days of therapy in such patients. However, following U.S. FDA recommendations this amendment was rescinded shortly after its implementation.

## Results

**Figure 1:**
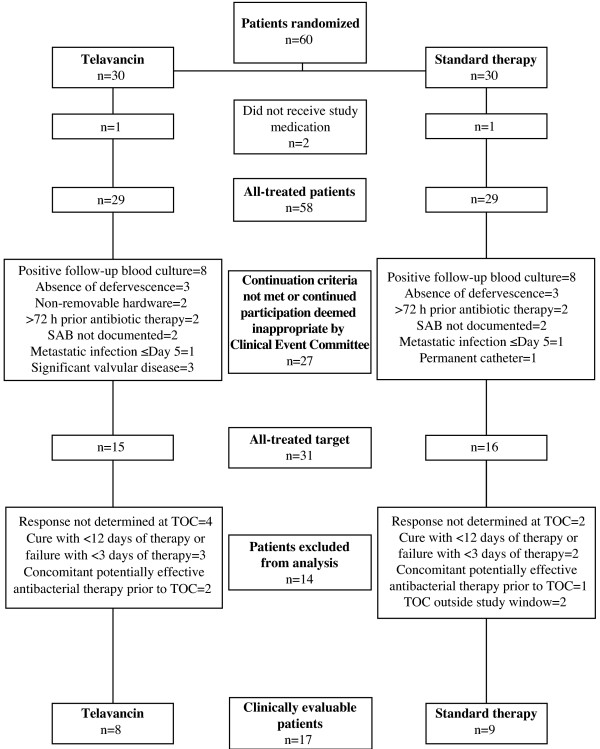
**Patient disposition into the study populations.** More than one reason may be present for patients to be excluded from all-treated target and/or clinically evaluable populations. SAB, *S. aureus* bacteremia; TOC, test of cure.

A total of 60 patients were randomized in the study. Among those, 58 patients from 21 sites in five countries received at least one dose of study medication (AT population) (Table 1). Patients were enrolled in the U.S., Argentina, Spain, Singapore, and Hong Kong. Most patients were enrolled in the U.S. (73%). Figure 1 displays the disposition of patients into the study populations. Only one patient was included under the amendment allowing complicated bacteremia, received telavancin 10 mg/kg q 24 h for 24 days, and was discontinued due to an AE.

**Table 1:** Study visits and reasons for early discontinuation of study medication

	**Telavancin**	**Standard therapy**	**Overall**
	**(n = 29)**	**(n = 29)**	**(n = 58)**
Completed FU visit	23 (79%)	26 (90%)	49 (84%)
Completed TOC visit	14 (48%)	17 (59%)	31 (53%)
Completed 14 days of therapy	13 (45%)	17 (59%)	30 (52%)
Discontinued study drugs early	16 (55%) ^*a*^	12 (41%)	28 (48%)
Reason for early study drug discontinuation			
Continuation criteria not met^*b*^	5 (17%)	2 (7%)	7 (12%)
Adverse event	2 (7%)	2 (7%)	4 (7%)
Consent withdrawal	2 (7%)	2 (7%)	4 (7%)
2 Consecutive ECGs with QTc >500 msec	0	1 (3%)	1 (2%)
Major protocol deviation	1 (3%) ^*c*^	0	1 (2%)
Other	6 (21%)	5 (17%)^*d*^	11 (19%)

*AE*, adverse event; *ECG*, electrocardiogram; *FU*, follow-up visit; *TOC*, test-of-cure visit.

^*a*^Includes the only patient who was included under the amendment for complicated bacteremia and who was discontinued due to an AE after receiving 24 days of therapy.

^*b*^A patient may have failed more than one continuation criterion; in patients failing continuation criteria the reason for early drug discontinuation decided by the investigators may have been different from not meeting such continuation criteria.

^*c*^This patient also failed continuation criteria.

^*d*^One patient in this group also failed continuation criteria.

A total of 55% of patients in the telavancin group and 41% in the standard therapy group discontinued the study drugs early. The most common reason for early discontinuation of study drug due to continuation criteria failure was the presence of positive FUBC. Among telavancin-treated patients who discontinued the drug early because of a positive FUBC result, four of five had their FUBC obtained before starting telavancin. Study visits and reasons for early study drug discontinuation are displayed in Table 1.

Overall, demographics and clinical characteristics were similar in the two groups (Table 2; ATT population). Most patients were male, white, and the mean age was 60 years. Diabetes was common in both treatment groups. There were more obese patients in the telavancin treatment group. The source of bacteremia was identified in the vast majority of cases, with IV catheters being the most common source in both groups. All IV catheters identified as a source of infection were removed within 24 h after the report of the qualifying blood culture. The majority of patients in the telavancin and standard therapy groups received prior systemic antimicrobial therapy with vancomycin within 7 days prior to initiation of study medication. MRSA was isolated from the blood cultures of 47% and 50% of the telavancin and standard therapy patient groups, respectively.

**Table 2:** Clinical and demographic characteristics in the all-treated target population

	**Telavancin**	**Standard therapy**	**Overall**
	**(n = 15)**	**(n = 16)**	**(n = 31)**
Age in years (mean ± SD)	59 ± 16.3	60 ± 20.4	60 ± 18.2
Age ≥65	5 (33%)	7 (44%)	12 (39%)
Gender, male	10 (67%)	8 (50%)	18 (58%)
Race, white	11 (73%)	10 (63%)	21 (68%)
Body mass index ≥30	6 (40%)	3 (19%)	9 (29%)
Diabetes	7 (47%)	10 (63%)	17 (55%)
Prior antimicrobial therapy^*a*^	14 (93%)	16 (100%)	30 (97%)
Vancomycin	13 (87%)	14 (88%)	27 (87%)
Primary source of bacteremia identified	12 (80%)	13 (81%)	25 (81%)
IV catheter	8 (53%)	9 (56%)	17 (55%)
Skin and soft tissue	3 (20%)	3 (19%)	6 (19%)
Other	1 (7%)	1 (6%)	2 (6%)
MRSA	7 (47%)	8 (50%)	15 (48%)

*IV*, intravenous; *MRSA*, methicillin-resistant *Staphylococcus aureus*.

^*a*^Within 7 days prior to initiation of study medication.

All baseline isolates of *S. aureus* available for testing were susceptible to vancomycin at a minimum inhibitory concentration (MIC) of ≤1 μg/ml (n = 40; MIC_90_ = 1 μg/ml for both MRSA and MSSA) and to telavancin at an MIC of ≤0.5 μg/ml (n = 32; MIC_90_ = 0.5 μg/ml for both MRSA and MSSA). Telavancin MICs for these isolates were tested according to the Clinical Laboratory Standards Institute (CLSI) guidelines in place at the time that this study was performed and susceptibility was interpreted with the corresponding FDA breakpoints approved in 2009. The MIC testing methodology and corresponding FDA-approved breakpoints for telavancin have recently been revised and are published in the CLSI M100-S24 guidelines and telavancin product insert (March 2014), respectively.

In the standard therapy group, seven of 16 patients were treated with an ASP from the beginning and one patient was switched from vancomycin to ASP on study day 4 due to a blood culture being positive for MSSA on day 3.

The CE population was limited to eight patients in the telavancin and nine patients in the standard therapy group. A similar proportion of patients were cured in the telavancin (88%) and standard therapy (89%) treatment groups (Table 3). All patients with MRSA bacteremia were cured, and one patient with *methicillin-resistant S. aureus* bacteremia failed study treatment in each treatment group (Table 4). The patient who failed therapy in the telavancin group was a 73-year-old female with a peripheral catheter-related MSSA bacteremia whose blood cultures were positive on study Day 28 and was subsequently found to have osteomyelitis. The patient who failed therapy in the standard therapy group was a 75-year-old male with MSSA bacteremia from an unidentified source who died after being readmitted on study Day 48 with an intestinal ischemia and positive blood cultures for MSSA.

**Table 3:** Cure rates and missing patients in the all-treated target and clinically evaluable populations

	**Telavancin**	**Standard therapy**	**95% CI for the difference (telavancin–standard therapy)**
	**n/N (%)**	**n/N (%)**	
All-treated target			
End-of-therapy visit	11/15 (73%)	15/16 (94%)	(–44.4, 7.8)
Follow-up visit	9/15 (60%)	14/16 (88%)	(–53.6, 4.5)
Test-of-cure visit	8/15 (53%)	11/16 (69%)	(–45.9, 18.5)
Clinically evaluable			
End-of-therapy visit	8/8 (100%)	9/9 (100%)	(–26.1, 24.3)
Follow-up visit	7/8 (88%)	9/9 (100%)	(–41.0, 19.1)
Test-of-cure visit	7/8 (88%)	8/9 (89%)	(–35.5, 31.9)
MRSA	5/5 (100%)	4/4 (100%)	-
MSSA	2/3 (67%)	4/5 (80%)	-

*CI*, confidence interval; *MRSA*, methicillin-resistant *Staphylococcus aureus*; *MSSA*, methicillin-susceptible *Staphylococcus aureus*.

**Table 4:** Main characteristics, therapy, and outcomes of patients with SAB (clinical evaluable population)

**Age, years, gender**	**Source of infection**	**Pathogen**	**Days of IV therapy**	**Agent**	**Microbiological response**	**Clinical response**
Telavancin, n *=* 8						
55, male	Peripherally inserted central catheter	MRSA	15	Telavancin	Eradication	Cure
55, male	Central IV catheter	MRSA	14	Telavancin	Eradication	Cure
74, male	Peripheral IV catheter	MRSA	14	Telavancin	Eradication	Cure
37, male	Central IV catheter	MRSA	14	Telavancin	Eradication	Cure
53, male	Cellulitis, associated with a previous peripheral IV catheter	MSSA	12	Telavancin	Eradication	Cure
63, female	Central IV catheter	MRSA	13	Telavancin	Eradication	Cure
56, female	Central IV catheter	MSSA	15	Telavancin	Eradication	Cure
73, female	Peripheral IV catheter	MSSA	15	Telavancin	Relapse	Failure
Standard therapy, n *=* 9						
25, female	Cellulitis	MRSA	15	Vancomycin	Relapse	Cure
59, male	Peripheral IV catheter	MRSA	14	Vancomycin	Eradication	Cure
87, female	Peripherally inserted central catheter	MSSA	14	Vancomycin	Eradication	Cure
75, male	N/A	MSSA	15	ASP	Relapse	Failure
55, female	Peripherally inserted central catheter	MSSA	15	Vancomycin	Eradication	Cure
83, female	Decubitus ulcer	MRSA	13	Vancomycin	Eradication	Cure
50, female	Central IV catheter	MSSA	15	ASP	Eradication	Cure
75, male	Central IV catheter	MSSA	15	ASP	Eradication	Cure
53, male	Central IV catheter	MRSA	13	Vancomycin	Eradication	Cure

*ASP*, anti-staphylococcal penicillin; *IV*, intravenous; *MRSA*, methicillin-resistant *Staphylococcus aureus*; *MSSA*, methicillin-susceptible *Staphylococcus aureus*; *N/A*, not available; *SAB*, *Staphylococcus aureus* bacteremia.

Among CE patients, microbiological eradication at TOC was achieved in 88% of patients in the telavancin group and 78% of patients in the standard therapy group. The two patients who relapsed (one in each treatment group) were considered clinical failures and have already been described. In addition, one patient in the standard therapy group who was considered clinically cured at TOC had one of two BCs positive for MRSA. The most relevant characteristics and outcomes of patients in the CE population are described in Table 4.

AEs and serious AEs (SAEs) were more common in the telavancin group (Table 5), although only two patients in each group discontinued the study medication because of an AE. Overall types of AEs were similar in the two study groups. Most common AEs included pyrexia, headache, anemia, and rash. No individual SAE occurred in more than one patient per treatment group. Five and three patients died in the telavancin and standard therapy groups (17% vs. 10%), respectively. Among patients who died in the telavancin group, one patient withdrew consent after the first dose and died from endocarditis, two patients were discontinued early because of failure to meet continuation criteria (one with endocarditis and one with metastatic soft tissue abscess), and two patients died after completing study medication (one with prostate cancer and cardiorespiratory failure and one who the investigator deemed to have [probable] sepsis from the urinary tract). Among patients who died in the standard therapy group, one patient died with MSSA bacteremia and intestinal ischemia (described above), one patient developed endocarditis, and one patient died from a neuroleptic malignant syndrome. Potential clinically significant increases in serum creatinine (serum creatinine ≥1.5 mg/dl and at least 50% greater than baseline at any time point through the EOT visit) were more common in telavancin-treated patients (5/25 vs. 2/28). The rates of resolution (completely or partially resolved) were two of five telavancin patients and two of two vancomycin patients by the last study visit. Otherwise, laboratory abnormalities were similar between the study groups (Table 6).

**Table 5:** Safety parameters in the all-treated population

	**Telavancin**	**Standard therapy**
	**(n = 29)**	**(n = 29)**
Deaths	5 (17%)	3 (10%)
Serious adverse events	11 (38%)	6 (21%)
Discontinuing study drug due to an adverse event	2 (7%)	2 (7%)
≥1 adverse event	26 (90%)	21 (72%)
Adverse event ≥5% in any treatment arm		
Pyrexia	4 (14%)	2 (7%)
Headache	3 (10%)	3 (10%)
Anemia	3 (10%)	2 (7%)
Rash^*a*^	2 (7%)	3 (10%)
Deep vein thrombosis	3 (10%)	1 (3%)
Hypokalemia	3 (10%)	1 (3%)
Nausea	1 (3%)	3 (10%)
Vomiting	1 (3%)	3 (10%)
Catheter site erythema	2 (7%)	1 (3%)
Dysgeusia^*b*^	3 (10%)	0
Agitation	2 (7%)	1 (3%)
Insomnia	2 (7%)	1 (3%)
Hematuria	1 (3%)	2 (7%)
Atelectasis	2 (7%)	1 (3%)
Dyspnea	1 (3%)	2 (7%)
Pruritus	1 (3%)	2 (7%)
Phlebitis	1 (3%)	2 (7%)
Urinary tract infection^*c*^	4 (14%)	0
Acute renal failure	2 (7%)	0
Blood urea increased	2 (7%)	0
Eosinophilia^*d*^	0	4 (14%)
Diarrhea	0	2 (7%)
Catheter site infection	0	2 (7%)

^*a*^Including adverse events termed “rash,” “maculo-papular rash,” and “rash pruritic.”

^*b*^Usually metallic taste.

^*c*^Including adverse events termed “urinary tract infection fungal” and “urinary tract infection.”

^*d*^Including adverse events termed “eosinophilia” and “eosinophil count increased.”

**Table 6:** **Laboratory abnormalities in all-treated patients with baseline normal values**^*a*^

	**Telavancin**	**Standard therapy**
	**n/N**	**n/N**
Hematocrit		
Male, ≤30%	1/4 (25%)	0/1 (0%)
Female, ≤28%	0/4 (0%)	0/3 (0%)
WBC ≤2800/μl	0/14 (0%)	0/15 (0%)
Platelet count ≤75,000/μl	0/18 (0%)	0/13 (0%)
AST (≥3 ULN)	3/18 (17%)	0/18 (0%)
ALT (≥3 ULN)	1/19 (5%)	1/17 (6%)
Alkaline phosphatase (≥1.5 ULN)	2/17 (12%)	1/22 (5%)
Potassium <3 meq/l	2/24 (8%)	0/19 (0%)
Potassium >5.5 meq/l	1/24 (4%)	3/19 (16%)
Creatinine increase^*b*^	5/25 (20%)	2/28 (7%)
Baseline creatinine <1.5 mg/dl	4/18 (22%)	0/20 (0%)
Baseline creatinine ≥1.5 mg/dl	1/7 (14%)	2/8 (25%)

*ALT*, alanine aminotransferase; *AST*, aspartate aminotransferase, *ULN*, upper limit of normal; *WBC*, white blood count.

^*a*^Includes laboratory assessments after initiation of study drug up to and including the earlier of the follow-up visit or 28 days after the last dose of study medication.

^*b*^Serum creatinine ≥1.5 mg/dl and at least 50% greater than baseline; includes patients with normal and abnormal values at baseline.

## Discussion

The ASSURE study is the first clinical trial evaluating telavancin in the treatment of patients with uncomplicated SAB. The study has provided several findings that should be noted.

This study provides proof-of-concept for telavancin in patients with uncomplicated SAB. Although cure rates in ATT patients were numerically higher (not statistically significant) in the standard therapy group, a similar proportion of CE patients were cured in the telavancin and standard therapy arm (88% vs. 89%) (Table 3). One patient in the standard therapy group who was considered cured had positive blood cultures during the FU visit. Although this patient should have been considered a clinical failure, the authors have chosen to be conservative in this report and maintain the original assessment. The only CE patient who failed in the telavancin group was found to have osteomyelitis several weeks after finishing therapy. While overall mortality of SAB in the pre-antibiotic era was over 80% [24], death rates still remain high (~30%) in contemporary series, particularly in patients with complicated disease [2,25,26]. The relatively low mortality found in this trial is consistent with both adequate antimicrobial therapy and selection of patients with uncomplicated disease.

Telavancin was tolerated in patients with uncomplicated SAB. Although AEs were more common in patients receiving telavancin, AEs leading to drug discontinuation were similar in the two groups. Consistent with a previously reported study, increases in serum creatinine were more common among telavancin-treated patients [27]. Changes in serum creatinine appeared reversible when an adequate FU was obtained and other contributing factors removed. Apparent differences in mortality observed in the AT population should be analyzed with caution. The apparent higher mortality observed in the telavancin group may be related to the small study size, and the fact that three died after being withdrawn early due to complicated disease and two died of other underlying conditions–urinary tract infection/sepsis and prostate cancer.

Clinical identifiers of complications in patients with SAB have been well described and include delayed clinical and microbiological response to antibacterial therapy [2]. Despite these observations, identifying patients with uncomplicated SAB at the initial evaluation is quite challenging. For example, in a study that identified clinical predictors of the presence of complicated *S. aureus* bacteremia among 724 prospectively identified patients, the risk for complications in patients with no identified risk factors was still ~16%. Moreover, the most powerful predictor of complicated *S. aureus* bacteremia, the presence of positive blood cultures at 48 to 96 h following the initial BC, was by definition unavailable at the time the patient was being considered for initial enrollment into the study [2]. Our inability to accurately define the extent of the disease at baseline in the present study of uncomplicated SAB resulted in a significant limitation in both enrollment and clinical evaluability. This also highlights the significant unmet medical and antimicrobial development need for rapid diagnostic platforms that can reduce the time to identification of the etiology and extent of bacterial bloodstream infection.

This study has several important limitations. First, the small study size limited the interpretation of outcome data. Although a larger study would provide more definitive evidence, the ASSURE trial provides proof-of-concept that telavancin could potentially be an effective therapy to treat patients with uncomplicated SAB and warrants further study. Second, the diagnostic techniques using blood cultures to determine bloodstream infection are still far from ideal. Hopefully, in the future, molecular techniques (e.g., polymerase chain reaction [PCR]) for rapid determination of SAB and antibiotic susceptibilities will be used in both clinical care and in trials of new antimicrobial agents [28]. Third, vancomycin was administered following local policies, and as such, adjustment according to trough levels was not mandatory. Although this fact may have resulted in inadequate dosing (high or low), only one patient with MRSA in the CE population experienced a late relapse, suggesting that vancomycin dosing was appropriate in most cases. There are also more recent recommendations on vancomycin dosing that were published after the start of this trial that will need to be considered in future studies. Lastly, the exclusion of patients with complicated disease resulted in a very small population of evaluable patients. There was also a possibility of underestimation of AEs due to the small sample size. In the absence of data from well-designed, adequately powered studies, physicians will continue to treat patients suffering from this life-threatening infection based on limited evidence on antibiotic efficacy.

## Conclusions

This study represents the first proof-of-concept for telavancin in patients with uncomplicated SAB. Based on these results, additional studies of telavancin in patients with SAB, including complicated SAB, are warranted.

## Endnote

^a^Telavancin is a lipoglycopeptide antibiotic approved in the United States and Canada for the treatment of patients with complicated skin and skin structure infections due to susceptible Gram-positive pathogens, and in the United States and Europe for the treatment of hospital-acquired bacterial pneumonia, including ventilator-associated bacterial pneumonia (HABP/VABP) due to susceptible isolates of *Staphylococcus aureus* (methicillin-resistant strains [MRSA] only in Europe), when alternative medicines are unsuitable.

## Abbreviations

AE: Adverse event; ASP: Anti-staphylococcal penicillin; AT: All-treated (population); ATT: All-treated target (population); BC: Blood culture; CE: Clinically evaluable (population); CI: Confidence interval; CSLI: Clinical Laboratory Standards Institute; EOT: End of therapy; FDA: U.S. Food and Drug Administration; FU: Follow-up; FUBC: Follow-up blood culture; hVISA: Hetero vancomycin intermediate susceptible *Staphylococcus aureus*; IV: Intravenous; QBC: Qualifying blood culture; MIC: Minimum inhibitory concentration; MRSA: Methicillin resistant *Staphylococcus aureus*; MSSA: Methicillin susceptible *Staphylococcus aureus*; PCR: Polymerase chain reaction; SAB: *Staphylococcus aureus* bacteremia; SAE: Serious adverse event; TOC: Test of cure.; VISA: Vancomycin intermediate susceptible *Staphylococcus aureus* .

## Competing interests

Martin E. Stryjewski has served as a consultant for Cempra, Cerexa, Furiex, Nabriva, PRA, The Medicines Company, Theravance, and Trius; has received grants from Duke University (NIH); and has received other financial support (including reimbursement for travel expenses and/or manuscript preparation) from Cempra, JMI Laboratories, and Theravance. Arnold Lentnek is an employee of Danbury Clinical Research and Infectious Disease Medical Practice of New York; has served as a consultant for Warburg Pincus Capital Investment; has received grants from Optimer; and has provided expert testimony for Sullivan Papain Block McGrath & Cannavo. William O’Riordan has received financial support related to being an ASSURE study investigator (including reimbursement for travel expenses) from Theravance. John Pullman has received grants and travel expenses reimbursement from Theravance; and has received payment for lectures/speakers bureaus from Astellas. Paul Anantharajah Tambyah has received grants from Sanofi-Pasteur and Theravance; has served as a consultant for AstraZeneca, GlaxoSmithKline, and Johnson & Johnson; has received payment for lectures/speakers bureaus from MSD and Novartis; has received payment for the development of educational presentations from Teleflex; and has received other financial support (including reimbursement for travel expenses) from 3M, Adamas, Fabentech, and Inviragen. Jose M. Miró has received financial support related to being an ASSURE study investigator from Theravance; has received grants from Cubist, Fondo de Investigaciones Sanitarias (Spanish Ministry of Health), National Institutes of Health, Novartis, and RIS-ISCIII (Spanish Network for AIDS Research, Instituto de Salud Carlos III); has participated on advisory boards for Cubist and Novartis; has served as a consultant for Abbott, Bristol-Myers Squibb, Cubist, Gilead Sciences, Merck, Novartis, Pfizer, Roche, and Theravance; has received payment for lectures/speakers bureaus from Abbott, Boehringer-Ingelheim, Bristol-Myers Squibb, Cubist, GlaxoSmithKline, Gilead Sciences, Janssen-Cilag, Merck Sharp & Dohme, Novartis, Pfizer, Roche, Schering-Plough, Theravance, and ViiV. Vance G. Fowler, Jr. has participated on advisory boards for Merck; has received grants from Advanced Liquid Logistics, Cerexa, Merck, MedImmune, National Institutes of Health, Novartis, Pfizer, and Theravance; has served as a consultant for Achaogen, Affinium, Astellas, Biosynexus, Cerexa, Durata, The Medicines Company, MedImmune, Novartis, Novadigm, Pfizer, and Theravance; is a patent holder by the National Center for Genomic Research; has received royalties from UpToDate; and has received payment for development of educational presentations from Cerexa, Cubist, and Theravance. Steven L. Barriere is an employee of, and holds equity securities of, Theravance. Michael M. Kitt was an employee of Theravance at the time of study conduct, and holds equity securities of Theravance. G. Ralph Corey has participated in advisory boards for Cempra, Cerexa, Inimex, Pfizer, and Trius; has served as a consultant for Cempra, Cerexa, Dr Reddy’s Lab, Inimex, PolyMedix, Pfizer, PRA, Theravance, and Trius; has received grants from Innocoll, The Medicines Company, and Theravance; and has received other financial support (including reimbursement for travel expenses and manuscript preparation) from Theravance.

## Authors' contributions

MES, AL, WO, JP, PAT, and JMM were study investigators. SLB and MMK were the sponsor medical monitors and were involved in the study concept/design and data analysis/interpretation. VGF was co-investigator and was involved in the study concept/design and data analysis/interpretation. GRC was the principle study investigator and was involved in the study concept/design and data analysis/interpretation. All authors were involved in preparation of the manuscript (via critical review) and approved the final version.

## Authors' information

Michael M Kitt, an employee of Theravance at the time of the study, has since left the company.

## Pre-publication history

The pre-publication history for this paper can be accessed here:

http://www.biomedcentral.com/1471-2334/14/289/prepub

## Supplementary Material

Additional file 1 IRB List ASSURE Study. Click here for file
