# Supplementary material for: A randomized Phase 2 trial of telavancin versus standard therapy in patients with uncomplicated Staphylococcus aureus bacteremia: the ASSURE study
Source: BMC Infect Dis. 2014 May 23;14:289. doi: 10.1186/1471-2334-14-289 (PMC4048626; doi:10.1186/1471-2334-14-289)
Supplement: Additional file 1 — IRB List ASSURE Study. [file 1471-2334-14-289-S1.docx]

**ADDITIONAL FILE ‒ IRB List ASSURE Study**

| **Country** | **Institutional Review Board/Ethics Committee** |
| --- | --- |
| Argentina | Comite de Revision Institucional Sanatorio Mitre  Bartolomé Mitre 2553  (C1039AAO) Ciudad de Buenos Aires    Comite de Etica en Investigacion Clinical  "Dr. Virgilio Foglia"  Tucuman 335-7° "D"  (C1049AAG) Ciudad de Buenos Aires |
| Argentina | Comite de Etica, Docencia, e Investigacion  Instituto de Investigaciones Medicas "Alfredo Lanari"  Avenida Combatientes de Malvinas 3150  (C1427ARN) Ciudad de Buenos Aires    Comite de Etica en Investigacion Clinical  "Dr. Virgilio Foglia"  Tucuman 335-7° "D"  (C1049AAG) Ciudad de Buenos Aires |
| Argentina | CEMIC Comite de Etica en Investigacion  Galvan 4102 (C1431FWN) Ciudad de Buenos Aires    Comite de Etica en Investigacion Clinical  "Dr. Virgilio Foglia"  Tucuman 335-7° "D"  (C1049AAG) Ciudad de Buenos Aires |
| Argentina | Comite de Etica Sanatorio Otamendi  Azcuenaga 870  (C1029AAP) Ciudad de Buenos Aires    Comite de Etica en Investigacion Clinical  "Dr. Virgilio Foglia"  Tucuman 335-7° "D"  (C1049AAG) Ciudad de Buenos Aires |
| Argentina | Comite de Docencia e Investigacion  Instituto Cardiovascular de Buenos Aires (ICBA)  Blanco Encalada 1543  (C1428DCO) Ciudad de Buenos Aires    Comite de Etica en Investigacion Clinical  "Dr. Virgilio Foglia"  Tucuman 335-7° "D"  (C1049AAG) Ciudad de Buenos Aires |
| Argentina | Comite de Docencia e Investigacion  Hospital General de Agudos Dr. Cosme Argerich  Almirante Brown 240  (C1155ADP) Ciudad de Buenos Aires  Comite de Bioetica  Hospital General de Agudos Dr. Cosme Argerich  Almirante Brown 240  (C1155ADP) Ciudad de Buenos Aires  Comite de Etica en Investigacion Clinical  "Dr. Virgilio Foglia"  Tucuman 335-7° "D"  (C1049AAG) Ciudad de Buenos Aires |
| Argentina | Comite Hospitalario de Bioetica  Hospital de Infecciosas Francisco Javier Muñíz  Uspallata 2272  (C1282AEN) Ciudad de Buenos Aires  Comite de Docencia e Investigacion  Hospital de Infecciosas Francisco Javier Muñíz  Uspallata 2272  (C1282AEN) Ciudad de Buenos Aires  Comite de Etica en Investigacion Clinical  "Dr. Virgilio Foglia"  Tucuman 335-7° "D"  (C1049AAG) Ciudad de Buenos Aires |
| Argentina | Comite de Docencia e Investigacion  Hospital Udaondo  Avenida Caseros 2061, 4° Piso  (C1264AAA) Ciudad de Buenos Aires  Comite de Bioetica del Hospital "Dr.Carlos Bonorino Udaondo"  Avenida Caseros 2061  (C1264AAA) Ciudad de Buenos Aires  Comite de Etica en Investigacion Clinical  "Dr. Virgilio Foglia"  Tucuman 335-7° "D"  (C1049AAG) Ciudad de Buenos Aires |
| France | CCPPRB de Franche Comte  CHU de Besançon  Hôpital Saint Jacques  25030 Besançon Cedex |
| Hong Kong | Ethics Subcommittee  Hong Kong East Cluster  Pamela Youde Nethersole Eastern Hospital  3 Lok Man Road, Chai Wan  Hong Kong |
| Singapore | NHG Domain Specified Review Board B  6 Commonwealth Lane  Level 6 GMTI Building  Singapore 149547  Clinical Trials Coordinating Committee  c/o Center for Drug Adminsitration  Health Sciences Authority  Block 1, 2 Jalan Bukit Merah  Singapore 169547 |
| Singapore | Singapore General Hospital  Institutional Review Board/Ethics Committee  Outram Road  Singapore 169608  Clinical Trials Coordinating Committee  c/o Center for Drug Adminsitration  Health Sciences Authority  Block 1, 2 Jalan Bukit Merah  Singapore 169547 |
| South Africa | Faculty of Health Sciences Research  Ethics Committee  University of Pretoria  Pretoria Academic Hospital  Soutpansberg Road,  MRC-Building, Level 2  Pretoria  Private Bag X385  Pretoria 0001 |
| South Africa | Prof JR Snyman  SAMAREC  The Chairman  SAMA Research Ethics Committee  PO Box 74789  Lynnwood Ridge 0040 |
| South Africa | Medunsa Research Ethics Committee  MEDUNSA  PO MEDUNSA  0204 |
|  |  |
| Spain | Comité Ético de Investigación Clínica  Hospital Clinic i Provincial de Barcelona  C/ Villarroel, 170  08036 Barcelona |
| Spain | Comité Ético de Investigación Clínica  Hospital Univeristario Virgen del Rocio  Avenida Manueal Siurot, s/n  41013 Sevilla |
| Spain | Comité Ético de Investigación Clínica  Fundación Jiménez Díaz  Instituto de investigaciones Médicas  Avenida Reyes Católicos n°2  28034 Madrid |
| Spain | Comité Ético de Investigación Clínica  Hospital Universitario Marqués de Valdecilla  Avendia Valdecilla s/n  39008 Santander |
| Spain | Comité Ético de Investigación Clínica  Hospital General Universitario Gregorio Marañon  C/ Doctor Escuerdo n° 46  28007 Madrid |
| Spain | Comité Ético de Investigación Clínica  Hospital Donostia  Paseo Dr. Berguiristain s/n  20014 Donostia  San Sebastián |
| Spain | Comité Ético de Investigación Clínica  Hospital de Bellvitge  Edificio de la Fundacion August Pi y Sunyer, Planta Baja  C/ Feixa Llarga s/n  08907Hospitalet de Llobregat  Barcelona |
|  |  |
|  |  |
|  |  |
|  |  |
|  |  |
| Spain | Comité Ético de Investigación Clínica Regional  Hospital Universitario Central de Asturias  C/ Celestino Villamil s/n  33006 Oviedo |
| Spain | Comité Ético de Investigación Clínica  Hospital General Vall d'Hebron  Pg. Vall d' Hebron 119-129  08035 Barcelona |
|  |  |
|  |  |
|  |  |
| Spain | Comité Ético de Investigación Clínica de la Fundación Hospital Alcorcón  Fundación Hospital Alcorcón  C/ Budapest n°1  28922 Alcorcón  Madrid |
|  |  |
| Spain | Comité Ético de Investigación Clínica del Hospital Universitario Reina Sofía  Hospital Universitario Reina Sofía  Avendia Menéndez Pidal s/n  14004 Córdoba |
|  |  |
|  |  |
|  |  |
|  |  |
| United States | Western Institutional Review Board  3535 7th Avenue Southwest  Olympia, Washington 98508 |
| [United States](mailto:woriordan@estudysite.com) | Forsyth Medical Center  Institutional Review Board  3333 Silas Creek Parkway  Winston-Salem, North Carolina 27103 |
| [United States](mailto:galia.rahav@sheba.health.gov.il) | William Beaumont Hospital  Human Investigation Committee  3811 West Thirteen Mile Road  Royal Oak, Michigan 48073 |
| [United States](mailto:kcasey@meridianhealth.com) | Durham Veterans Administration Institutional Review Board  508 Fulton Street  Durham, North Carolina 27705 |
| [United States](mailto:kcasey@meridianhealth.com) | Fox Commercial Institutional Review Board  326 North Seventh Street, Suite 218  Springfield, Illinois 62701 |
| United States | University of Wisconsin-Madison  Health Sciences Human Subjects Committee  2500 Overlook Terrace, Room B3088  Madison, Wisconsin 53705 |
| [United States](mailto:cohl@wfubmc.edu) | Jersey Shore Medical Center  Institutional Review Board  1945 Route 33  Neptune, New Jersey 07754 |
| United States | Inova Institutional Review Board  Inova Fairfax Hospital  3300 Gallows Road  Falls Church, Virginia 22042 |
| [United States](mailto:gvalainis@srhs.com) | University of Mississippi Institutional Review Board  2500 North State Street  Jackson, Mississippi 39216 |
| United States | Institutional Review Board Paradise Valley Hospital  2400 East Fourth Street  National City, California 91950    Western Institutional Review Board  3535 7th Avenue Southwest  Olympia, Washington 98508 |
| United States | East Tennessee State University/Veterans Administration  Institutional Review Board  Box 70565  East Tennessee State University  Johnson City, Tennessee 37614 |
|  |  |
|  |  |
|  |  |
|  |  |
| United States | Spartanburg Regional Medical Center  Institutional Review Board  101 East Wood Street  Spartanburg, South Carolina 29303 |
| United States | University South Florida  Office of Research, Division of Research Compliance  12901 Bruce B. Downs Boulevard, MDC035  Tampa, Florida 33612 |
| United States | Lehigh Valley Hospital Institutional Review Board  Health Studies Research  Seventeenth and Chew Streets  Sixth Floor, #38  Allentown, Pennsylvania 18104 |
| United States | Sharp HealthCare  Office for the Protection of Research Participants  8695 Spectrum Center Boulevard  San Diego, California 92123 |
| United States | Saint Mary's Medical Center Institutional Review Board  901 Forty-fifth Street  West Palm Beach, Florida 33401 |
| United States | Iowa Health - Des Moines  Institutional Review Board  1215 Pleasant Street, Suite 305  Des Moines, Iowa 50309 |
| United States | Tulane University Health Sciences Center  Committee on the Use of Human Subjects  1430 Tulane Avenue  New Orleans, Louisiana 70112 |
| United States | Edward Hospital and Health Services  Institutional Review Board  801 South Washington Street  Naperville, Illinois 60540 |
| United States | Carle Institutional Review Board  611 West Park Street - MERC  Urbana, Illinois 61801 |
| United States | Hackensack University Medical Center  Institutional Review Board  30 Prospect Avenue  Hackensack, New Jersey 07601 |
| United States | Summa Health System Hospitals Institutional Review Board  525 East Market Street  Akron, Ohio 44309 |
| United States | Human Studies Subcommittee  Research Service (151)  Central Texas Veterans Health Care System  1901 Veterans Memorial Drive  Temple, Texas 76504 |
| United States | Duke University Health System  Institutional Review Board  Suite 8300 North Pavilion  Box 2991  Durham, North Carolina 27710 |
| United States | Our Lady of Resurrection Medical Center  Institutional Review Board  5645 West Addison Street  Chicago, Illinois 60634 |
| United States | Human Studies Subcommittee  Veterans Administration New Jersey Health Care System  385 Tremont Avenue  East Orange, New Jersey 07018 |
| United States | Monmouth Medical Center Institutional Review Board  Department of Academic Affairs  300 Second Avenue, Stanley 223  Long Branch, New Jersey 07740 |
| United States | Ochsner Clinic Foundation Institutional Review Board  1514 Jefferson Highway  New Orleans, Louisiana 70121 |
| United States | Western Institutional Review Board  3535 7th Avenue Southwest  Olympia, Washington 98508 |
| United States | Western Maryland Health System  Institutional Review Board  900 Seton Drive  Cumberland, Maryland 21502 |
| United States | Wake Forest University Health Sciences  Institutional Review Board  Medical Center Boulevard  Winston-Salem, North Carolina 27157-1023 |
| United States | University of Maryland Baltimore  Institutional Review Board  685 West Baltimore Street  Baltimore, Maryland 21201 |
| United States | Wayne State University  Human Investigation Committee  4201 Saint Antoine Boulevard, UHC-6G  Detroit, Michigan 48201 |
| United States | Human Subjects Committee  Los Angeles Biomedical Research Institute at Harbor-UCLA Medical Center  1124 West Carson Street, N-14  Torrance, California 90502 |
| United States | Henry Ford Hospital  Institutional Review Board  2799 West Grand Boulevard  CFP-Basement  Detroit, Michigan 48202 |
